# Draft genomic and transcriptome resources for marine chelicerate *Tachypleus tridentatus*

**DOI:** 10.1038/sdata.2019.29

**Published:** 2019-02-26

**Authors:** Yong Yan Liao, Peng Wei Xu, Kit Yue Kwan, Zhi Yun Ma, Huai Yi Fang, Jun Yang Xu, Peng Liang Wang, Shao Yu Yang, Shang Bo Xie, Shu Qing Xu, Dan Qian, Wei Feng Li, Li Rong Bai, Da Jie Zhou, Yan Qiu Zhang, Juan Lei, Ke Liu, Fan Li, Jian Li, Peng Zhu, Yu Jun Wang, Hai Ping Wu, You Hou Xu, Hu Huang, Chi Zhang, Jin Xia Liu, Jun Feng Han

**Affiliations:** 1 Guangxi Key Laboratory of Beibu Gulf Marine Biodiversity Conservation, Qinzhou, 53501 Guangxi, China; 2 Ocean College, Beibu Gluf University, Qinzhou, 535011, Guangxi, China; 3 BGI Genomics, BGI-Shenzhen, 518083, Shenzhen, China

**Keywords:** Conservation genomics, Genome

## Abstract

Chinese horseshoe crabs (*Tachypleus tridentatus*), ancient marine arthropods dating back to the mid-Palaeozoic Era, have provided valuable resources for the detection of bacterial or fungal contamination. However, excessive exploitation for the amoebocyte lysate of *Tachypleus* has dramatically decreased the population of the Chinese horseshoe crabs. Thus, we present sequencing, assembly and annotation of *T. tridentatus,* with the hope of understanding the genomic feature of the living fossil and assisting scientists with the protection of this endangered species. The final genome contained a total size of 1.943 Gb, covering 90.23% of the estimated genome size. The transcriptome of three larval stages was constructed to investigate the candidate gene involved in the larval development and validate annotation. The completeness of the genome and gene models was estimated by BUSCO, reaching 96.2% and 95.4%, respectively. The synonymous substitution distribution of paralogues revealed that *T. tridentatus* had undergone two rounds of whole-genome duplication. All genomic and transcriptome data have been deposited in public databases, ready to be used by researchers working on horseshoe crabs.

## Background & Summary

Horseshoe crabs in the world are represented by two extant populations following a particular geographical distribution^1^. *Tachypleus tridentatus* (2n = 26), *Tachypleus gigas* (2n = 28) and *Carcinoscorpius rotundicauda* (2n = 32) inhabit the Asian coastline of Southeast Asia; *Limulus polyphemus* (2n = 52) is distributed along the Atlantic coastline of North America^2^. Horseshoe crabs have the rare feature of providing blue blood, which has extensive infection-fighting properties that can be used to quantify gram-negative bacterial endotoxins or fungal contamination of medical products^3^. The horseshoe crabs show some features of crustaceans (crab shell and claws), but they are not crabs at all and are more closely related to spiders, scorpions and trilobites^4,5^. They belong to their own class called Merostomata, which shows three divisions of the body: the prosoma, opisthosoma and telson.

*T. tridentatus*, the tri-spine horseshoe crab, the largest of the living horseshoe crab species, was once widespread along the coast of Fujian, Hainan and Beibu Gulf Bay^2^, but now, the population has drastically decreased for various reasons, such as environmental pollution, decreasing coastline and excessive exploitation for blood extracts (amoebocyte lysate). *T. tridentatus* is now a ‘Grade II Protected Animal of China’ and is ‘protected aquatic wildlife’ in the Fujian and Guangxi Zhuang Autonomous Region. Although many horseshoe crab nature reserves have been established since 2001 in Guangdong Province around the northern South China Sea, more marine reserve networks for horseshoe crabs in the South and East China Seaboards are needed to establish with hope for protection for the habitat and recovery of this population of the *T. tridentatus*^6^.

The external morphology of horseshoe crabs has remained nearly unchanged since the Ordovician approximately 445 million years ago^7^. The morphological resemblance of a modern horseshoe crab to its ancient fossil suggests the question of whether the genome of the horseshoe crab has been as slow to evolve as the horseshoe crab’s outward appearance implies. Although several genome or transcriptome projects have been implemented on the horseshoe crabs with genotyping-by-sequencing or low-depth sequencing strategies, such as provided in the draft genome of the Atlantic horseshoe crab genome (*L. polyphemus*)^8–10^, the partial genome of three horseshoe crabs (*C. rotundicauda*, *L. polyphemus* and *T. tridentatus*)^11^, and the *de novo* transcriptome of *T. tridentatus* during embryonic development^12^, the lack of fully sequenced and finely annotated horseshoe crab genomic information has hindered the emergence of arthropod models for slow evolution, which has been widely discussed in the brachiopod *Lingula anatina*^13^ and the Sarcopterygii *Latimeria chalumnae*^14^.

To understand the genomic feature of the living fossil and protect these endangered species, we have generated a draft genome and transcriptome of *T. tridentatus*. We extracted genomic DNA from leg muscle tissues, constructed 4 Paired-End (PE) and 5 Mate-Pair (MP) libraries, which were subsequently sequenced using the Illumina HiSeq 2500 or Hiseq 4000 platforms. Three stages (pre-trilobite, trilobite, and post-trilobite) of larva, containing two biological replicates for each stage, were chosen for further transcriptome sequencing. After filtering out the low quality, adaptor-contaminated or PCR duplication reads of genome sequencing, we applied the KMERFREQ_AR v2.0.4^15^ and JELLYFISH v1.1^16^ to evaluate the genome size, heterozygosis and repetition by calculating the frequency of 17-mers and then used the Platanus v1.2.4^17^ to assemble the genome using all clean reads from PE and MP libraries. Another round of gap-filling steps was performed based on the assembly results, utilizing GapCloser v1.12-r6^15^. The final assembled genome size was 1.94 Gb, containing 736,826 contigs (N50 = 52.2 kb) and 671,877 scaffolds (N50 = 2.76 Mb), which represented approximately 90.16% of the genome estimated via *k*-mer analysis. The BUSCO v2.1^18^ and *de novo* assembled transcript evaluation demonstrated the genome was of considerable completeness and high gene region coverage. The transposable elements accounted for 39.96% of the *T. tridentatus* genome, whereas the DNA transposons accounted for the largest portion, 23.06%. The structural annotation of the genome yielded 29,134 genes, and approximately 83.37% of these genes were functionally annotated with at least one of four sources (InterPro, KEGG, SwissProt and TrEMBL). The synonymous substitution (Ks) distribution of the *T. tridentatus* paralogues indicated the Chinese horseshoe crabs had undergone two rounds of WGDs, as has its Atlantic counterpart. The release of the *T. tridentatus* genome and transcriptome in this study provides a crucial resource for future efforts to adopt better strategies to conserve the endangered Asian horseshoe crab and to take it as the model species to foster study on marine chelicerates.

## Methods

### Tissue sampling

**Figure 1:**
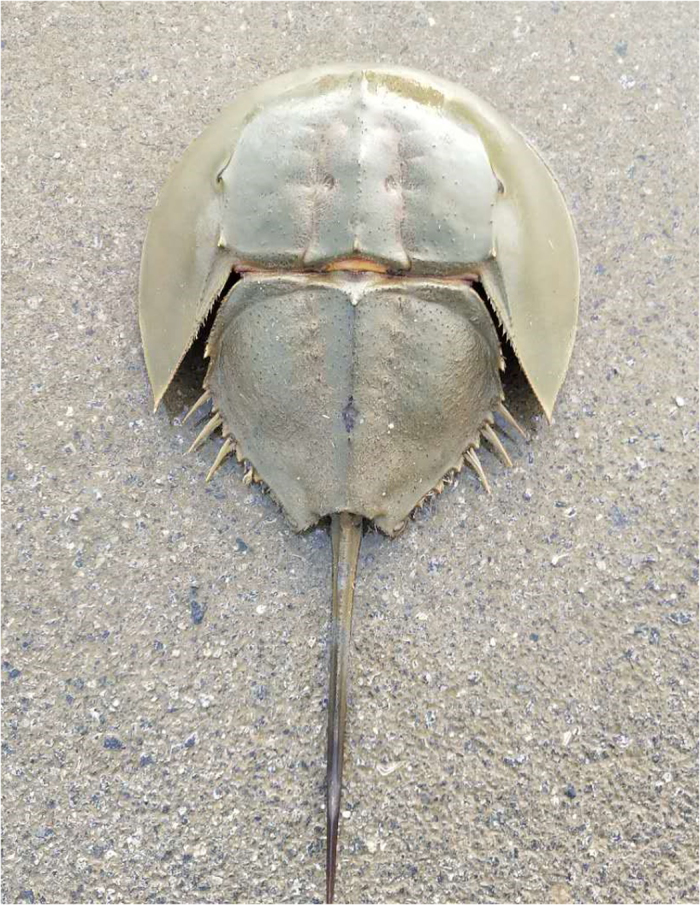
The morphology of *T. tridentatus*.

For genomic sequencing, an adult *T. tridentatus* (Fig. 1) was acquired from the Guangxi Key Laboratory of Beibu Gulf Marine Biodiversity Conservation. Leg skeletal muscle tissue was collected and then stored in liquid nitrogen for immediate DNA extraction. For transcriptome sequencing, six fertilized eggs were collected by laboratory spawning and incubated with standard procedures under appropriate temperature and salinity^19^. Three larval stages were collected according to Sekiguchi’s definition^20^: ‘pre-trilobite’, ‘trilobite’ and ‘post-trilobite’. Two biological replicates for each larval stage were collected and stored in liquid nitrogen for immediate RNA extraction.

### Library construction and sequence quality control

Genomic DNA was extracted from frozen muscle tissues of *T. tridentatus* using a genomic DNA isolation kit (Qiagen, Hilden, Germany) and the manufacturer’s protocol and then stored at −80 °C until library preparation. Subsequently, 4 pair-end libraries with insert sizes of 270 bp, 300 bp, 500 bp and 800 bp, and 5 mate-pair libraries with insert sizes of 2 kb, 5 kb, 10 kb, 20 kb and 40 kb were generated (Data Citation 1), sequenced by BGI (Shen Zhen) on Illumina Hiseq 2500 or Hiseq 4000, following the manufacturer’s instruction. Finally, we generated a total of 554.2 Gb raw bases from 19 lanes (Table 1). The raw reads of the PE and MP libraries were then filtered by SOAPnuke v1.5^21^ according to the following criteria: 1) reads with adapter contamination or polymerase chain reaction duplicates were discarded, 2) reads with more than 30% low-quality bases (Q value ≤ 15) for the pair-end reads and more than 20% low-quality bases (Q value ≤ 10) for the mate-pair reads were removed, 3) reads with more than 1% N bases were removed, and 4) matching lengths of read 1 and read 2 were removed when 10 bp had at least a 10% mismatch. After sequences had been pre-processed with SOAPnuke v1.5 software, a total of 202.67 Gb clean pair-end and 71.54 Gb mate-pair reads were obtained (Table 1).

The total RNA of each larval development was extracted separately according to the TRIzol protocol (Invitrogen). Samples were sequenced by BGI (Shen Zhen) on an Illumina HiSeq 2500 platform (Data Citation 1). Paired-end reads were generated with a read length of 100 bp. A total of 436.63 Mb RNA-Seq reads were produced (Table 2). The raw reads of the transcriptome libraries were filtered by SOAPnuke v1.5 according to the following criteria: 1) reads with more than 1% N bases were removed; 2) reads with more than 20% low-quality bases (Q value ≤ 10) were removed; and 3) reads with adapter contamination were discarded. After pre-processing with SOAPnuke software, a total of 393.57 Mb RNA-seq reads were generated with an average Q20 ≥ 96% (Table 2).

### Characteristics of the *T. tridentatus* genome

**Figure 2:**
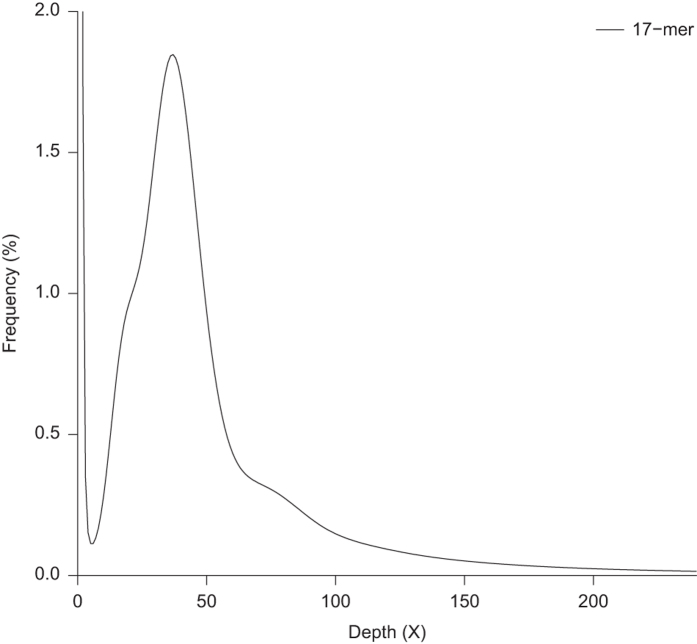
K-mer distribution used for the estimation of genome size. The distribution was determined with KMERFREQ_AR using a k-mer size of 17.

A total of 91.34 Gb clean reads from libraries for insert sizes of 300 bp and 500 bp were chosen for use in *k*-mer analysis using KMERFREQ_AR v2.0.4^15^ and JELLYFISH v1.1^16^ with a k length of 17. The genome size (G) of *T. tridentatus* was estimated by the following formula: G = *k*-mer number/*k*-mer depth, where the k-mer number is the total numbers of *k*-mers, and *k*-mer depth refers to the depths of the main peak in the *k*-mer frequency distribution. The *k*-mer frequency distribution indicated that *T. tridentatus* was a diploid species with low heterozygosity and repetition (Fig. 2). The frequency of 17-mers using KMERFREQ_AR v2.0.4^15^ estimated a genome size of 2.15 Gb, whereas the JELLYFISH v1.1^16^ approach yielded an estimated genome size of 2.14 Gb (Supplementary Table 1). The result of JELLYFISH was subsequently delivered to GenomeScope^22^, indicating the heterozygosity of the *T. tridentatus* genome was approximately 0.65%. The estimated genome size of *T. tridentatus* is much smaller than *L. polyphemus*, which is estimated to be 2.74 Gb (2.8 pg) based on the biochemical method^23^.

### Genome assembly

For genome assembly, the contig construction, scaffold connection and gap-closer of the *T. tridentatus* genome was performed based on multiple *k*-mer values automatically optimized by the Platanus v1.2.4^17^, with clean reads from pair-end and mate-pair libraries. All applications were used with the default parameters except for the initial *k*-mer 37. Furthermore, one more round of gap-closer was performed using GapCloser v1.12-r6^15^ to fill the remaining gaps in the scaffolds. The final assembly contained 671,877 scaffolds and included a total length of 1.94 Gb, which represented approximate 90.16% of the genome estimated by the *k*-mer analysis. The sizes of the longest scaffold and contig were 18,230,544 and 1,165,240 bp, respectively, and 87.92% of the assembled sequences with lengths longer than 10 kb were among the 2,573 scaffolds. The contig and scaffold N50s were 52,179 and 2,761,313 bp, respectively, whereas the percentage of the gap in the genome was 1.55% (Table 3).

### Transcriptome assembly

The *de novo* transcriptome assembly of each larval stage was performed using Trinity v2.4.0^24^ with default parameters. The Trinity assemble resulted in 669,788 transcripts with an average N50 ~1 Kb (Supplementary Table 2). The transcriptome assembly of the same stage was then clustered to remove redundancies and form the Unigenes using the TGI Clustering Tool (TGICL) v2.1^25^ with default parameters. The TGICL cluster generated 273,085 transcripts with an average N50 ~1.5 Kb (Supplementary Table 2). The genome-guided transcriptome assembly of each larval stage was performed with HiSat2 v2.2.1.0^26,27^ and StringTie v1.3.4^27,28^. The genome index was built using HISAT2-build, and then, the clean transcriptome reads were mapped to the genome using HiSat2, and the alignment result of each larval stage was merged to form one single BAM file using SAMtools v1.3^29^. Finally, the genome-guided transcriptome assembly was performed using StringTie with the single BAM file.

### Repeat annotation

Prior to gene prediction, the transposable elements were identified in the genome of *T. tridentatus.* The transposable elements were identified by a combination of homology and ab initio-based methods. For the homology method, RepeatMaskerv4.0.5^30^ was adopted to identify the transposable elements against the RepBase library^31^ (release-20170127), while RepeatProteinMask v4.05^30^ was applied to identify the transposable elements against the TE protein databases attached with RepBase library. For the ab initio-based method, the *de novo* library of *T. tridentatus* genome was constructed using LTR_FINDER v1.07^32^ and REPEATMODELER v1.0.8^33^. Subsequently, RepeatMasker v4.05 was used to identify and classify different categories of repetitive elements against the *de novo* library. Finally, the transposable elements of the same category identified by these two methods were integrated via sequence overlap. The percentage of the *T. tridentatus* genome covered by transposable elements was 39.96%, with a total length of 776,373,528 bp; meanwhile, the DNA transposons took up the largest portion— 23.06% (Supplementary Table 3).

### Gene prediction

The MAKER^34^ application was used to predict the gene model by integrating the homology, transcriptome and ab initio gene predictions. MAKER was initially run in the est2genome = 1 and protein2genome = 1 model, which created a gene model directly from the transcript and protein evidence. The transcript evidence was based on *de novo* transcriptome assembly of the three larval stages, which included 273,085 transcripts (Supplementary Table 2), whereas the 96,006 protein sequences were collected from the genome of four arthropods deposited at NCBI: *Limulus polyphemus*^10,35^, *Mesobuthus martensii*^36^, *Stegodyphus mimosarum*^37^ and *Ixodes scapularis*^38^. The option ‘rm_gff’ was filled out with the transposable element file acquired from the repeat annotation step, and the expected max intron size for evidence alignments was set to 30,000 according to the max intron size of *L. polyphemus*. The initial run produced 25,252 gene models, and 2,000 high-confidence gene models were randomly selected according to the criterion determined by the maker2zff (an application in MAKER pipeline) default parameters, except for the maximum annotation edit distance (AED) of 0.1. These high confidence gene models were then used to train the parameters of SNAP^39^ (release-2013-11-29) and AUGUSTUS v3.3.1^40^ software with each pipeline. A second round of MAKER was run with the training parameters, est2genome = 0 and protein2genome = 0 mode. All other parameters were the same as the first round except that we used ‘est_gff’ with the genome guiding transcriptome assembly to replace the ‘est’ option.

### Gene function annotation

Protein sequences from the predicted gene models were searched against the KEGG^41^ (v84.0), SwissProt and TrEMBL databases^42^ (release2017-09) with E-value threshold of 1e-5. The domain annotations were applied using InterProScan^43^ (5.16-55.0) and by searching against the public databases Pfam^44^, PRINTS^45^, ProDom^46^, PIRSF^47^, PANTHER^48^, TIGRFAM^49^, SUPERFAMILY^50^, ProSitePatterns^51^, ProSiteProfiles^52^, Coils^53^ and SMART^54^. Approximately 83.37% of these genes were functionally annotated with at least one of these sources, with 21,414 InterProScan entries, 19,195 KEGG entries, 15,283 SwissProt entries and 23,687 TrEMBL entries (Supplementary Table 4).

### Whole-genome duplications in *L. polyphemus* and *T. tridentatus*

The synonymous substitutions (Ks) distribution had been used to infer WGD in plant and vertebrates^55,56^. To inspect the evidence for the whole-gene duplication of the Chinese and Atlantic horseshoe crabs, we identified the paralogues of each species and calculated the substitutions per synonymous site (Ks) distribution of paralogous pairs with a Python script available online: https://github.com/EndymionCooper/KSPlotting. The main steps were listed as follows. 1) Sequence similarity was determined using an all-vs-all comparison of protein sequences, performed using BLASTP + v2.50^57^ with an E-value threshold of 1e-5. The paired genes were retained under the criterion that the shorter sequence was at least 50% of the longer sequence and that the alignment length was at least 50% of the shorter sequence. 2) Paralogue gene family construction was determined by the paralogue gene families that were built through single-linkage clustering. In brief, the genes with multiple alignment and associated matches were grouped into the same paralogue categories. After the paralogues had been identified, all possible pairs of protein sequences in each paralogue family were aligned using MUSCLE^58^ v3.8.31 with default parameters, and then the multiple alignments of the amino acid sequences were converted to the corresponding coding sequences (CDS). 3) Synonymous substitutions (Ks) values of each paired paralogue gene were calculated using CODEML^59^. Only gene pairs with a Ks estimate of <3 were considered for further analysis. A paralogous gene family of n members was derived from n-1 possible duplication events, but the number of probable pairwise Ks comparisons within a family was n × (n-1)/2, which could result in misconception of the ages of duplication events. The Ks values of each paralogous group were corrected to remove redundancy using a hierarchical clustering approach^60,61^, leaving the paralogous groups that contained the representative duplication events. We also retrieved all-vs-all alignment using the reciprocal best blast hit (RBH) criterion to identify the orthologous genes, and then, we applied KaKs Calculator^62^ with the method of Yang and Nielsen^63^ to estimate synonymous substitution rates (Ks) of the orthologs.

### Code availability

The software versions, settings and parameters are described below.

1) KMERFREQ_AR: version 2.04, k-mer size of 17; 2) JELLYFISH: version 1.1, k-mer size 17; 3) GenomeScope: parameters used were k-mer length 17; read length 100; maximum k-mer coverage 1000; 4) Platanus: version 1.2.4, parameters used were contig Platanus contig -t 20 -k 37 -s 10 -u 0.1 -d 0.5 -m 400 -f <insert size 270 bp pair-end reads> <insert size 300 bp pair-end reads> <insert size 500 bp pair-end reads> <insert size 800 bp pair-end reads> ; scaffold -u 0.1 -c contig.fa -b contigBubble.fa -IP1 <insert size 270 bp pair-end reads> -IP2 <insert size 300 bp pair-end reads> -IP3 <insert size 500 bp pair-end reads> -IP4 <insert size 800 bp pair-end reads> -OP5 <insert size 2 k mate-pair reads> -OP6 <insert size 5 k mate-pair reads> -OP7 <insert size 10 k mate-pair reads> -OP8 <insert size 10 k mate-pair reads> -OP9 <insert size 20 k mate-pair reads> -OP10 <insert size 40 k mate-pair reads> -a1 270 -a2 300 -a3 500 -a4 800 -a5 2000 -a6 5000 -a7 10000 -a8 10000 -a9 20000 -a10 40000; gap_close -c scaffold.fa -IP1 <insert size 270 bp pair-end reads> -IP2 <insert size 300 bp pair-end reads> -IP3 <insert size 500 bp pair-end reads> -IP4 <insert size 800 bp pair-end reads>; 5) Gap Closer: version 1.12, parameters used were -l 150, in configFile: asm_flags = 4; PE125 lib: rd_len_cutoff = 100, pair_num_cutoff = 7, map_len = 45; PE150 libs: rd_len_cutoff = 135, pair_num_cutoff = 10, map_len = 50; 6) BUSCO: version 2.1, arthropod default parameters, arthropoda_odb9; 7) SOAPnuke: version 1.5, genome sequences pre-processing: pair-end library: -l 15 -q 0.3 -n 0.1 -d -i -S; mate-pair library: -l 10 -q 0.2 -n 0.1 -d -i -S; RNA-Seq sequences pre-processing: -l 10 -q 0.2 -n 0.1 -i; 8) Trinity: version 2.4.0, default parameters; 9) TGICL: version 2.1, -l 40 -c 10 -v 25 -O ‘-repeat_stringency 0.95 -minmatch 35 -minscore 35’; 10) HiSat2: version 2.2.1.0, --max-intronlen50000 --sensitive --dta --dta-cufflinks --phred64 --no-mixed --no-discordant; 11) StringTie: version 1.3.4, default parameters; 12) SAMtools: version 1.3, default parameters; 13) RepeatMasker: version 4.0.5 (with RepBase library release-20170127); 14) RepeatModeler: RepeatModeler-open-1.0.8; 15) LTR_FINDER: version 1.07; 16) MAKER: version 2.3.10, first run, parameters: est = merged transcripts from Trinity assemblies for 3 larval stages, protein = 4 arthropod proteins, rmlib = all transposon elements, est2genome = 1, protein2genome = 1; predicted 25,252 genes, randomly select 2,000 to train Augustus and SNAP. Second run: est_gff = merged transcripts gff from StringTie assemblies for three larval stages, protein = 4 arthropod proteins, rmlib = all transposon elements, est2genome = 0, protein2genome = 0, snaphmm = SNAP training parameter, augustus_species = AUGUSTUS training parameter; 17) SNAP: release-2013-11-29; 18) AUGUSTUS: version 3.3.1; 18) KEGG: version 84.0; 19) SwissProt and TrEMBL: release 2017-09; 20) InterProscan: version 5.16-55.0, with parameters -goterms -f tsv -appl ProDom -appl PRINTS-appl Pfam -appl PIRSF -appl PANTHER -appl TIGRFAM -appl SUPERFAMILY -appl ProSitePatterns -appl ProSiteProfiles -appl Coils -appl SMART; 20) BLASTP: version 2.6.0; 21) MUSCLE: v3.8.31; 22) CODEML version 4.8; 23) KaKs Calculator version 2.0.

## Data Records

The raw data of the whole genome and the RNA-seq sequencing was submitted to the National Center for Biotechnology Information (NCBI) (Data Citations 1), and more detailed information about the reads is shown in Data_Descriptor_Worksheets.xlsx. The final assembly was deposited at NCBI GenBank (Data Citations 2). The other files, such as the assembled contigs, scaffold, *de novo* RNA-seq assembly, repeat annotation, gene prediction and gene function annotation were uploaded to Figshare (Data Citations 3), and the file with descriptions is presented in Supplementary Table 5.

## Technical Validation

### Genome assembly and gene prediction quality assessment

The completeness of the genome assembly and gene prediction was assessed using BUSCO^18^ based on evolutionarily informed expectations of gene content from near-universal single-copy orthologues selected from OrthoDBv9^64^. The completeness of the resulting assembly was comparable to the other arthropods; BUSCO analysis showed that 96.2 and 0.8% of the 1066 arthropod datasets (arthropoda_odb9, http://busco.ezlab.org/) were identified as complete and fragmented separately, whereas 3% of the BUSCO genes were missing from the assembled genome sequence (Supplementary Table 6). The completeness of the current gene prediction is also comparable to the other arthropod, 95.4% and 2.3% of the 1066 arthropod datasets (arthropoda_odb9, http://busco.ezlab.org/) were identified as complete and fragmented separately, whereas 2.3% of the BUSCO genes were missing from the current annotation (Supplementary Table 7). The *de novo* transcriptome assembly of three larval stages was aligned to the genome using BLAT^65^ with default parameters to evaluate the coverage of the gene region. The alignment results of the Unigenes indicated that the assembled genome of *T. tridentatus* covered 95.59~98.01% of the Unigenes, 89.02~93.55% of the Unigenes with at least 90% coverage in one scaffold, and 93.62~98.81% of the Unigenes with at least 50% coverage in one scaffold (Supplementary Table 8), suggesting that the gene regions were mostly included in the current assembly.

### Comparison with other horseshoe crab genomes

**Figure 3:**
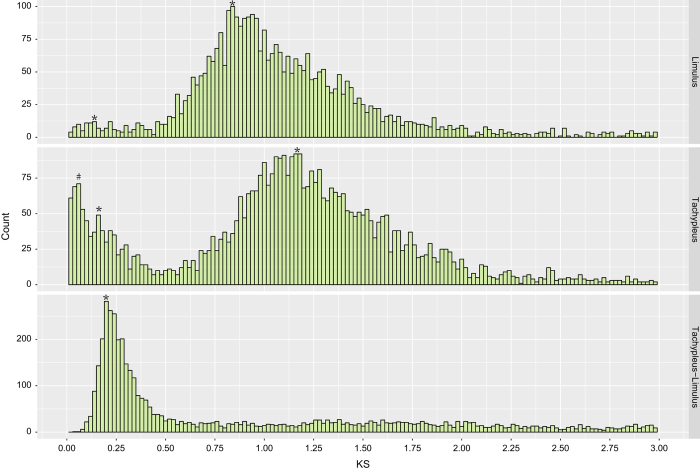
The synonymous substitutions (Ks) distribution of the paralogues and orthologues of the Chinese and Atlantic horseshoe crab. The ‘*’ indicates the peak derived from the whole-genome duplication event, whereas the ‘#’ indicates the peak derived from small-scale duplications events (SSDs, tandem duplications) in the Chinese horseshoe crab.

The current assembly of the *T. tridentatus* genome may be the most complete version of horseshoe crab. The contig and scaffold N50 of the current assembly were much longer than any other published horseshoe crab databases (Supplementary Table 9), the sequence depths and assembled sizes were also much larger than other sources. The longest scaffold of *T. tridentatus* measures 18,230,544 bp, which is three times more than *L. polyphemus* (PRJNA20489). The numbers of ≥1 Mb scaffold in *T. tridentatus* are 446, while the numbers of ≥ 1 Mb scaffold in *L. polyphemus* only are 147 (Table 3). The Ks distribution of *L. polyphemus* exhibited peak centred at 0.14 and 0.84, whereas the Ks distribution of *T. tridentatus* showed peaks centred at 0.16 and 1.16 (Fig. 3). The evidence showed the lineage of the horseshoe crabs had undergone two rounds of WGDs, which was consistent with the previous result^9^ but with a variant peak. Meanwhile, the Ks distribution of the orthologous genes between *T. tridentatus* and *L. polyphemus* showed a peak centred at 0.20 (Fig. 3), which suggests the last common ancestor of the extant horseshoe carb was posterior to the ancient duplication but predated the recent duplication.

## Usage notes

All analyses were run on Linux systems, and the optimal parameters are given in the Code availability section.

## Additional information

**How to cite this article**: Liao, Y. Y. *et al*. Draft genomic and transcriptome resources for marine chelicerate *Tachypleus tridentatus.* *Sci. Data*. 6:190029 https://doi.org/10.1038/sdata.2019.29 (2019).

**Publisher’s note**: Springer Nature remains neutral with regard to jurisdictional claims in published maps and institutional affiliations.

## Supplementary Material



Supplementary Information

## Figures and Tables

**Table 1:** Sequencing libraries and data yields for whole-genome shotgun sequencing.

Library type	Lane	platform	Read Length (bp)	Insert Size (bp)	Raw bases (Gb)	Clean bases (Gb)
PE150	1	Hiseq4000	150	270	42.25	39.4
PE125	1	Hiseq2500	125	300	79.72	58.9
PE125	1	Hiseq2500	125	500	78.01	59.58
PE125	1	Hiseq2500	125	800	59.34	44.79
MP49	3	Hiseq4000	49	2000	50.04	18.3
MP49	4	Hiseq4000	49	5000	53.65	20.41
MP49	2	Hiseq4000	49	10000	61.28	14.29
MP49	3	Hiseq4000	49	20000	66.15	9.63
MP49	3	Hiseq4000	49	40000	63.77	8.91
Total	19				554.21	274.21
Note: PE: paired-end, MP: mate pair. The raw reads were filtered using SOAPnuke.						

**Table 2:** RNA-Seq data yields of three larval stages.

Accession	Stage of larvae	Sample	Raw reads numbers (Mb)	Clean reads numbers (Mb)	Clean reads Q20
SRR7239295	pre-trilobite	TaL-1-1	67.87	66.14	96.79
SRR7239308	pre-trilobite	TaL-1-2	76.92	66.13	96.58
SRR7239307	trilobite	TaL-2-1	76.92	65.18	97.01
SRR7239306	trilobite	TaL-2-2	79.18	65.64	96.81
SRR7239305	post-trilobite	TaL-3-1	67.87	65.24	97.12
SRR7239304	post-trilobite	TaL-3-2	67.87	65.24	98.92
Total			436.63	393.57	
Note: The three developmental stages of larva were collected according to Sekiguchi’ s definition, “pre-trilobite”, “trilobite” and “post-trilobite”. Two biological replicates for each stage. The raw reads were filtered using SOAPnuke.					

**Table 3:** The statistic of *T. tridentatus* and *L. polyphemus* genome assembly.

	*T. tridentatus*				*L. polyphemus*^1^			
	Contig		Scaffold		Contig		Scaffold	
	Numbers	Sizes	Numbers	Sizes	Numbers	Sizes	Numbers	Sizes
Minimum length		4		41		200		200
Maximum length		1,165,240		18,230,544		133,547		5,191,289
Total Numbers	736,826		671,877		469,510		286,793	
N50	9,200	52,179	186	2,761,313	41,759	11,441	1,712	254,089
Total Size (bp)		1,912,885,564		1,942,936,674		1,705,786,612		1,828,271,751
>=1kp	96,184	1,761,273,298	45,307	1,797,749,641	222,804	1,592,186,184	50,802	1,722,826,540
>=2kb	62,482	1,715,053,174	15,879	1,757,753,852	165,672	1,511,153,688	24,654	1,687,761,575
>10Kb	34,272	1,584,470,853	2,573	1,708,312,084	49,849	939,425,146	13,197	1,641,492,664
>=100kb	3,139	528,273,461	1,150	1,666,730,451	9	996,662	4,168	1,307,731,143
>=1Mb	3	3,275,645	446	1,399,495,467	0	0	147	215,133,244
Gap sizes (bp)				30,051,110				122,485,139
Note: The statistical result of the genome assembly, the contig length is the genome assembly don’t contain ‘N’base.1: PRJNA20489, Washington University (WashU) submit.								
