# Author Correction: Draft genomic and transcriptome resources for marine chelicerate *Tachypleus tridentatus*

**DOI:** 10.1038/s41597-019-0025-6

**Published:** 2019-03-26

**Authors:** Yong Yan Liao, Peng Wei Xu, Kit Yue Kwan, Zhi Yun Ma, Huai Yi Fang, Jun Yang Xu, Peng Liang Wang, Shao Yu Yang, Shang Bo Xie, Shu Qing Xu, Dan Qian, Wei Feng Li, Li Rong Bai, Da Jie Zhou, Yan Qiu Zhang, Juan Lei, Ke Liu, Fan Li, Jian Li, Peng Zhu, Yu Jun Wang, Hai Ping Wu, You Hou Xu, Hu Huang, Chi Zhang, Jin Xia Liu, Jun Feng Han

**Affiliations:** 1 Guangxi Key Laboratory of Beibu Gulf Marine Biodiversity Conservation, Qinzhou, 53501 Guangxi China; 2 Ocean College, Beibu Gulf University, Qinzhou, 535011 Guangxi China; 3 BGI Genomics, BGI-Shenzhen, 518083 Shenzhen, China

Correction to: *Scientific Data* 6, 190029 10.1038/sdata.2019.29 published online 26 February 2019

In the original version of this Data Descriptor the word “Gulf” was incorrectly spelled in the affiliation “Ocean College, Beibu Gulf University, Qinzhou, 535011, Guangxi, China”. This has now been corrected in both the HTML and PDF versions.

