# Supplementary material for: Draft genomic and transcriptome resources for marine chelicerate Tachypleus tridentatus
Source: Sci Data. 2019 Feb 26;6:190029. doi: 10.1038/sdata.2019.29 (PMC6390705; doi:10.1038/sdata.2019.29)
Supplement: Supplementary Information [file sdata201929-s2.pdf]

Supplementary Table 1 *Tachypleus tridentatus* genome size estimated with KMERFREQ_AR and JELLYFISH using a k-mer size of 17 2

Supplementary Table 2 The *de novo* assembly of *Tachypleus tridentatus* transcriptome 2

Supplementary Table 3 Transposable elements in the *Tachypleus tridentatus* genome 2

Supplementary Table 4 Function annotation of the 29,134 predicted transcripts 2

Supplementary Table 5 Datasets submitted to the *Figshare* website and its data descriptions. 3

Supplementary Table 6 The comparative analysis of assembly results of five Arthropoda species with BUSCO software 4

Supplementary Table 7 The comparative analysis of gene prediction of five Arthropoda species with BUSCO software 4

Supplementary Table 8 The gene coverage based on three larval stages transcriptome 4

Supplementary Table 9 Characteristics of horseshoe crab assemblies available in public database 5

Supplementary Table 1 *Tachypleus tridentatus* genome size estimated with KMERFREQ_AR and JELLYFISH using a k-mer size of 17

Genome	K-mer length(bp)	K-mer numbers	Peak depths	Estimated Genome size	Read bases	Genome Coverage (X)
T. tridentatus[a]	17	79382418055	37	2145470758	91340804750	42.57
T. tridentatus[b]	17	79334690118	37	2144180814	91340804750	42.60

Note: a. The genome size estimated by KMERFREQ_AR; b. The genome size estimated by JELLYFISH

Supplementary Table 2 The *de novo* assembly of *Tachypleus tridentatus* transcriptome

Sample	Stage	Original Numbers	Original N50	Final Numbers	Final N50
TaL-1-1	Pre-trilobites	133,347	1,637	99,673	2,158
TaL-1-2	Pre-trilobites	132,953	1,523		
TaL-2-1	Trilobites	95,871	1,211	73273	1,650
TaL-2-2	Trilobites	89,594	1,126		
TaL-3-1	Post-trilobites	122,456	1,388	100,139	1,569
TaL-3-2	Post-trilobites	95,567	1,049		
Total		669,788		273,085	

Note: Original Numbers and N50 was the transcriptome result was assembled by Trinity v2.4.0. The Final Numbers and N50 was the Trinity assemblies of the same stage were clustered by TGICLv2.1.

Supplementary Table 3 Transposable elements in the *Tachypleus tridentatus* genome

	Repabse TEs length	Protein TEs length	*De novo* TEs length	Combined TEs	
				length	percentage (%)
DNA	69,235,790	51,944,659	433,216,221	448,039,818	23.059929
LINE	21,895,117	45,238,890	200,410,281	212,515,638	10.937857
SINE	4,725,513	0	105,470,215	108,648,204	5.591958
LTR	6,700,866	2,141,679	117,138,422	122,354,068	6.297378
Other	2,972	0	0	2,972	0.000153
Unknown	0	0	23,991,350	23,991,350	1.234798
Total	100,990,715	99,310,536	761,657,129	776,373,528	39.958766

Note: RepabseTEs means RepeatMask against Repbase; Protein TEs means RepeatProteinMask result against Repbase protein; *De novo* TEs means RepeatMask against the *de novo* library; Combined TEs means the combine result of three steps.

Supplementary Table 4 Function annotation of the 29,134 predicted transcripts

		Number	Percent (%)
Total gene numbers		29,134	
Annotated	InterPro	21,414	73.50
	KEGG	19,195	65.89
	SwissProt	15,283	52.46
	TrEMBL	23,687	81.30
Overall		24,289	83.37

Supplementary Table 5 Datasets submitted to the *Figshare* website and its data descriptions.

File name	type	Description
Genome Contigs/scaffolds/gapcloser		
Tachypleus_tridentatus.contig.fa.gz	fasta	platanus v1.2.4 contig step
Tachypleus_tridentatus.scaffold.fa.gz	fasta	platanus v1.2.4 scaffold step
Tachypleus_tridentatus.gapcloser.fa.gz	fasta	platanus v1.2.4 gapcloser step
Tachypleus_tridentatus.genome.fa.gz	fasta	Gapcloser v1.12-r6 assembly
Repeat		
Denovo_repeat_library.fa.gz	fasta	the denovo library constructed by RepeatModler and LTR_Finder
Tachypleus.denovo.RepeatMasker.gff.gz	gff3	*de novo* repeat elements of RepeatMasker output against the denovo library constructed by RepeatModler and LTR_Finder
Tachypleus.known.RepeatMasker.gff.gz	gff3	known repeat elements of RepeatMaker output against the known library archived in RepeatBase
Tachypleus.known.RepeatProteinMask.gff.gz	gff3	known repeat elements of RepeatProteinMasker output against the TE protein
Gene prediction		
Tachypleus_tridentatus.gene.cds.fa	fasta	coding gene nucleotide sequences (fasta)
Tachypleus_tridentatus.gene.protein.fa	fasta	coding gene translated sequences (protein fasta)
Tachypleus_tridentatus.gene.gff	gff3	coding gene annotations (gff format)
Function annotation		
Tachypleus_tridentatus.gene.iprscan.xls	xls	gene function annotation by interproscan-5.11-55.0
Tachypleus_tridentatus.KEGG.xls	xls	the gene annotation with best hit result of KEGG database
Tachypleus_tridentatus.Swissprot.xls	xls	gene annotation with best hit result of SwissProt database
Tachypleus_tridentatus.Trembl.xls	xls	gene annotation with best hit result of TrEMBL database
De novo assembly		
TaL-1-1A.Trinity.fa.gz	fasta	The trinity assembly of TAL-1-1 (Pre-trilobite)
TaL-1-2A.Trinity.fa.gz	fasta	The trinity assembly of TAL-1-2 (Pre-trilobite)
TaL-2-1A.Trinity.fa.gz	fasta	The trinity assembly of TAL-2-1 (Trilobite)
TaL-2-2A.Trinity.fa.gz	fasta	The trinity assembly of TAL-2-2 (Trilobite)
TaL-3-1A.Trinity.fa.gz	fasta	The trinity assembly of TAL-3-1 (Post-trilobite)
TaL-3-2A.Trinity.fa.gz	fasta	The trinity assembly of TAL-3-2 (Post-trilobite)
Pre-trilobites-stage.fa.gz	fasta	The TGICL cluster assembly of Pre-trilobite stage
Trilobites-stage.fa.gz	fasta	The TGICL cluster assembly of Trilobite stage
Post-trilobites-stage.fa.gz	fasta	The TGICL cluster assembly of Post-trilobite stage

Supplementary Table 6 The comparative analysis of assembly results of five Arthropoda species with BUSCO software

	Tachypleus tridentatus		*Limulus polyphemus*		*Ixodes capularis*		*Stegodyphus mimosarum*		*Tetranychus urticae*	
BUSCO	Ns	P (%)	Ns	P (%)	Ns	P (%)	Ns	P (%)	Ns	P (%)
C	1025	96.2	968	90.8	936	87.8	982	92.1	986	92.5
S	830	77.9	797	74.8	925	86.8	949	89.0	931	87.3
D	195	18.3	171	16.0	11	1.0	33	3.1	55	5.2
F	8	0.8	54	5.1	75	7.0	28	2.6	16	1.5
M	33	3	44	4.1	55	5.2	56	5.3	64	6.0

Note: Total BUSCO groups searched are 1066; Ns: numbers; P: Percent; C: Complete BUSCOs; S: Complete and single-copy BUSCOs; D: Complete and duplicated BUSCOs; F: Fragmented BUSCOs; M: Missing BUSCOs. Complete BUSCOs=Complete and single-copy BUSCOs + Complete and duplicated BUSCOs

Supplementary Table 7 The comparative analysis of gene prediction of five Arthropoda species with BUSCO software

	Tachypleus tridentatus		*Limulus polyphemus*		*Ixodes capularis*		*Stegodyphus mimosarum*		*Tetranychus urticae*	
BUSCO	Ns	P (%)	Ns	P (%)	Ns	P (%)	Ns	P (%)	Ns	P (%)
C	1017	95.4	1010	94.7	908	85.2	916	85.9	1000	93.9
S	832	78.0	806	75.6	890	83.5	884	82.9	928	87.1
D	185	17.4	204	19.1	18	1.7	32	3.0	72	6.8
F	25	2.3	41	3.8	106	9.9	121	11.4	12	1.1
M	24	2.3	15	1.5	52	4.9	29	2.7	54	5.0

Note: Total BUSCO groups searched are 1066; Ns: numbers; P: Percent; C: Complete BUSCOs, Complete BUSCOs = Complete and single-copy BUSCOs + Complete and duplicated BUSCOs; S: Complete and single-copy BUSCOs; D: Complete and duplicated BUSCOs; F: Fragmented BUSCOs; M: Missing BUSCOs.

Supplementary Table 8 The gene coverage based on three larval stages transcriptome

Dataset	Number of Unigenes	Total length (bp)	Base coverage by assembly (%)	>90% sequence in one scaffold (%)	>50% sequence in one scaffold (%)
Pre-trilobites	99,673	102,549,504	98.01	93.23	98.81
Trilobites	73,273	62,940,655	97.90	93.55	98.35
Post-trilobites	100,139	83,785,200	95.59	89.02	93.62

Note: Trinity assemblies of the same larval stage were clustered by TGICLv2.1

Supplementary Table 9 Characteristics of horseshoe crab assemblies available in public database

Species	Sequencing technology	Sequence coverage	Estimated Size (Gb)	Assembly Size (Gb)	Contig N50 (Kb)	Scaffold N50 (Kb)
Limulus polyphemus[1]	Roche 454<br>HiSeq2000	16x<br>12x	2.74[a]	1.88	11.44	254.09
Limulus polyphemus[2]	HiSeq2000	1.1x/per sample	2.74[a]	1.23	0.40	2.93
Limulus polyphemus[3]	Hiseq2000	23x	2.74[a]	1.48	0.47	
Limulus polyphemus[4]	HiSeq2000	5.8x	2.74[a]	1.45	0.45	0.47
Carcinoscorpius rotundicauda[4]	HiSeq2000	9.52x	2.74[a]	1.58	1.26	1.71
Tachypleus tridentatus[4]	HiSeq2000	12.14x	2.15[b]	1.53	0.55	0.59
Tachypleustridentatus[5]	HiSeq2500<br>HiSeq4000	127.54x	2.15[b]	1.94	52.18	2757.46

Note: data source 1. PRJNA20489, Washington University (WashU) submit; 2. PRJNA187356, Rice University submit; 3. PRJNA340394, University of New Hampshire submit; 4. PRJNA243016, Chinese University of Hong Kong submit; 5. PRJNA472682, Qinzhou University provide; a: *Limulus polyphemus* genome size estimated based on biochemical analysis; b. The genome size estimated by KMERFREQ_AR with 91.34 Gb paired-end reads
